# An Unusual Case of Unilateral Papilledema

**DOI:** 10.5811/cpcem.2019.8.43461

**Published:** 2019-10-21

**Authors:** Daniel Quesada, Matthew Stapleton, Jadipak Heer, Phillip Aguìñiga-Navarrete, Luke Kim

**Affiliations:** * Kern Medical Center, Department of Emergency Medicine, Bakersfield, California; † LA+USC Medical Center, Department of Emergency Medicine, Los Angeles, California

## Abstract

Neuroretinitis from neurosyphilis is an uncommon finding in previously healthy young individuals. A 37-year-old presented with three days of painless, unilateral vision loss with an associated diffuse erythematous non-pruritic truncal rash. Physical exam demonstrated vision loss in the left eye. Fundoscopic exam showed unilateral peripapillary hemorrhage, papilledema and venous engorgement. Labs showed positive syphilis antibody qualitative. Magnetic resonance imaging demonstrated 12 millimeters of high right frontal lobe cerebrospinal fluid density. The patient was treated with benzylpenicillin and within 18 hours had improvement of his vision.

## CASE PRESENTATION

**Image 1:**
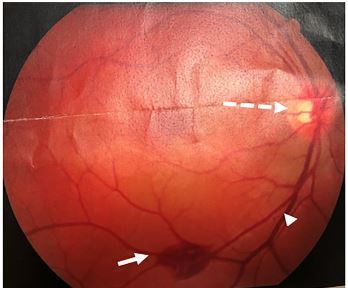
Fundoscopic findings revealing peripapillary hemorrhage (white arrow), papilledema (dashed arrow), and venous engorgement (arrowhead).

A 37-year-old male presented to the emergency department complaining of three days of painless left eye vision changes. He described the changes as a “white out.” He also noted a four-week-old diffuse, erythematous, nonpruritic truncal rash. Visual exam findings were notable for oculus sinister of 20/25. Oculus dexter was 20/20. There was no presence of ptosis. Bilateral fluorescein stain and slit lamp exam were unremarkable. A fundoscopic exam of the left eye revealed unilateral papilledema (UP) and bilateral retinal hemorrhage (Image 1). Ocular pressures were unremarkable.

Labs were significant for reactive hepatitis B, antinuclear antibody screen, rapid plasma reagin test with reflex, fluorescent treponemal antibody absorption test, sedimentation rate Westergren automated test, and c-reactive protein of 3.1 milligrams per deciliter (mg/dL) (0.0–3.1 mg/dL). Venereal disease research laboratory on cerebrospinal fluid (CSF) was nonreactive. Lyme antibody screen and Bartonella antibody panel were both negative. Syphilis antibody qualitative was positive. Rapid human immunodeficiency virus (HIV) test was negative. Syphilitic and UP findings prompted a lumbar puncture to rule out neurosyphilis, which subsequently revealed elevated lymphocytes 100% (40–80%) and protein 65.0 mg/dL (15.0–45.0 mg/dL).

**Image 2:**
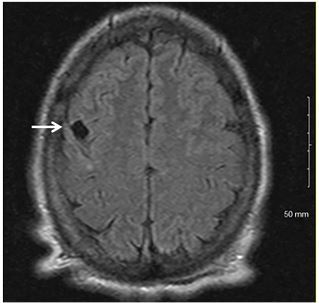
Magnetic resonance image of the brain revealing 12-millimeter right frontal lobe cerebrospinal fluid density (arrow).

A magnetic resonance imaging of the brain revealed 12 millimeters of high right frontal lobe CSF density (Image 2). The patient was given benzylpenicillin with subsequent vision improvement within 18 hours of administration, indicative of painless vision loss secondary to neurosyphilitic neuroretinitis.

## DISCUSSION

Common causes of UP and vision loss include anterior ischemic optic neuropathy (AION) and optic neuritis (ON).^1^ AION is often seen in patients older than 50 years with associated comorbidities, making it an unlikely cause of this patient’s vision loss.^1^ ON typically affects females between 20–35 years of age.^1^ ON in 90% of cases has associated headaches, eye pain or both, whereas 19% of AION cases have associated pain.^1^ Neuroretinitis is uncommon, typically characterized by optic disc edema and subsequent formation of a macular star figure. The underlying pathophysiology involves increased permeability of disc vasculature, but is not fully defined.^2^

Our patient’s exam and symptoms were most consistent with neuroretinitis. Most cases of neuroretinitis are reported in ophthalmology literature and in association with cat scratch disease (CSD). Only about 1% of the 12,000 yearly cases of CSD present with neuroretinitis. Those who have reported cases of ophthalmological complaints not neuroretinitis specifically are individuals who are HIV positive.^3^ Our specific case demonstrates an uncommon presentation of unilateral papilledema in a healthy patient. Painless unilateral papilledema in younger patients should raise concern for an insidious process and prompt thorough investigation.

**CPC-EM Capsule**

What do we already know about this clinical entity?

*Neuroretinitis is most commonly found in patients with cat scratch disease*.

What is the major impact of the image(s)?

*This case demonstrates a rare presentation of unilateral papilledema in an otherwise healthy patient*.

How might this improve emergency medicine practice?

*Unilateral papilledema in younger populations presenting without pain should prompt a thorough medical and physical examination with a widened differential diagnosis*.
